# Investigating receptor-mediated antibody transcytosis using blood–brain barrier organoid arrays

**DOI:** 10.1186/s12987-021-00276-x

**Published:** 2021-09-20

**Authors:** Claire Simonneau, Martina Duschmalé, Alina Gavrilov, Nathalie Brandenberg, Sylke Hoehnel, Camilla Ceroni, Evodie Lassalle, Elena Kassianidou, Hendrik Knoetgen, Jens Niewoehner, Roberto Villaseñor

**Affiliations:** 1 Roche Pharma Research and Early Development (pRED), Pharmaceutical Sciences, Roche Innovation Center Basel, Basel, Switzerland; 2 SUN bioscience, EPFL Innovation Park, Lausanne, Switzerland; 3 Roche Pharma Research and Early Development (pRED), Therapeutic Modalities, Roche Innovation Center Munich, Munich, Germany

**Keywords:** Blood–brain barrier, Organoids, Receptor-mediated transcytosis, High-throughput in vitro model

## Abstract

**Background:**

The pathways that control protein transport across the blood–brain barrier (BBB) remain poorly characterized. Despite great advances in recapitulating the human BBB in vitro, current models are not suitable for systematic analysis of the molecular mechanisms of antibody transport. The gaps in our mechanistic understanding of antibody transcytosis hinder new therapeutic delivery strategy development.

**Methods:**

We applied a novel bioengineering approach to generate human BBB organoids by the self-assembly of astrocytes, pericytes and brain endothelial cells with unprecedented throughput and reproducibility using micro patterned hydrogels. We designed a semi-automated and scalable imaging assay to measure receptor-mediated transcytosis of antibodies. Finally, we developed a workflow to use CRISPR/Cas9 gene editing in BBB organoid arrays to knock out regulators of endocytosis specifically in brain endothelial cells in order to dissect the molecular mechanisms of receptor-mediated transcytosis.

**Results:**

BBB organoid arrays allowed the simultaneous growth of more than 3000 homogenous organoids per individual experiment in a highly reproducible manner. BBB organoid arrays showed low permeability to macromolecules and prevented transport of human non-targeting antibodies. In contrast, a monovalent antibody targeting the human transferrin receptor underwent dose- and time-dependent transcytosis in organoids. Using CRISPR/Cas9 gene editing in BBB organoid arrays, we showed that clathrin, but not caveolin, is required for transferrin receptor-dependent transcytosis.

**Conclusions:**

Human BBB organoid arrays are a robust high-throughput platform that can be used to discover new mechanisms of receptor-mediated antibody transcytosis. The implementation of this platform during early stages of drug discovery can accelerate the development of new brain delivery technologies.

**Supplementary Information:**

The online version contains supplementary material available at 10.1186/s12987-021-00276-x.

## Background

In recent years, development of technology platforms to deliver biologics to the brain parenchyma from the circulation has accelerated. Robust evidence from independent groups shows that targeting receptors present on endothelial cells at the blood–brain barrier (BBB) can lead to enhanced brain uptake of therapeutic proteins in preclinical species [1–5]. The diversity of shuttle formats (e.g. bispecific antibodies [1], single domain antibodies [6–9], Fc-engineered binders [3]) together with different receptor targets in brain endothelial cells (e.g. transferrin receptor [2, 10–12], insulin receptor [13], CD98 [14], TMEM30 [15]) expand the design space for successful brain delivery in a combinatorial manner. Despite these rapid advances in protein delivery technologies to the central nervous system (CNS), the understanding of the precise molecular mechanisms of receptor-mediated antibody transport across the BBB is lagging.

Recent studies have shed light on candidate pathways for antibody transcytosis across the BBB. We previously found that intracellular endosome tubules regulate transferrin receptor-based Brain Shuttle sorting for transcytosis [16]. Sorting tubules are also involved during transcytosis of the low-density lipoprotein receptor–related protein 1 [17] and a specific serotype of adeno-associated virus (AAV9) [18]. In contrast, FC5 transcytosis depends on multivesicular body transport [15]. These results highlight that receptor-mediated transcytosis may occur via a variety of independent pathways. Identifying the molecular regulators of these transport pathways requires functional genomic tools to evaluate the effect of individual genes on transcytosis, as was recently shown for polarized epithelial cells [19]. However, most work on the regulation of transcytosis at the BBB is still performed in rodents [20–22]. While these landmark efforts identified novel regulators for specific pathways, such as Mfsd2a [20] and ALPL [23], in vivo studies do not enable sufficiently high throughput and are not optimal to perform unbiased system-level analysis of transport mechanisms.

Multiple groups have recently established a novel 3D model of the BBB formed by the self-organization of human brain endothelial cells, pericytes and astrocytes [24–27]. Self-assembled 3D BBB models allow direct cell–cell interactions in the absence of artificial membranes or substrates and, importantly, recapitulate key cellular and molecular properties of the BBB, including: (1) tight junction formation, (2) efflux pump expression and activity and (3) receptor-mediated transport of peptides [26]. Since these 3D cellular assemblies undergo self-organization to display functional and morphological features of the BBB in vivo, they fulfill the criteria to be defined as organoids [28, 29]. Although BBB organoids are a major advance in the field of in vitro BBB modelling, organoid production, processing and analysis still require extensive manual work [30]. Thus, the complexity of the protocol and its relative low-throughput hinder its widespread adoption for screening purposes.

Here, we used a recently developed technology based on hydrogel micro-scaffolds [31] to generate patterned BBB organoid arrays, which allowed the formation of more than 3000 viable organoids with reproducible sizes in a 96-well plate format. In agreement with previously described human in vitro models, BBB organoid arrays restricted the passage of large molecules to the organoid core but recapitulated receptor-mediated transcytosis of a transferrin receptor-targeting antibody shuttle. Finally, we showed that BBB organoids can be combined with CRISPR/Cas9 gene editing to knock out specific genes in brain endothelial cells to evaluate their role in receptor-mediated transcytosis. Together, our data show that BBB organoid arrays are a high-throughput platform to discover new mechanisms of antibody transport across the BBB.

## Methods

### Fabrication of hydrogel-based U-bottom microwell arrays

U-bottom microwell hydrogel-based arrays (Gri3D, SUN bioscience) were fabricated and conditioned as previously described [31]. For the generation of high-throughput BBB organoids, microcavities of 600 μm in diameter and 720 μm in depth imprinted in polyethylene glycol (PEG) hydrogels (Elastic modulus: G’ = 12.5 kPa) were typically used.

### Culture conditions

Primary human astrocytes (HA, ScienCell Research Laboratories) were grown in Astrocyte Growth Medium (AGM) composed of Astrocyte Medium (AM, ScienCell Research Laboratories) supplemented with 2% FBS, 1% Astrocytes Growth Supplement (AGS, ScienCell Research Laboratories) and 1% penicillin/streptomycin. Human brain microvascular pericytes (HBVP, ScienCell Research Laboratories) were cultured in Pericyte Growth Medium (PGM) composed of Pericytes Medium (PM, ScienCell Research Laboratories) supplemented with 2% FBS, 1% Pericytes Growth Supplement (PGS, ScienCell Research Laboratories) and 1% penicillin/streptomycin. Human cerebral microvascular endothelial cells (hCMEC/D3, Sigma-Aldrich) were maintained in culture using Endothelial Basal Medium (EBM-2, Lonza) supplemented with hydrocortisone, GA-1000, 2% FBS, hEGF, VEGF, hFGF-B, R3-IGF-1, ascorbic acid and heparin (EGM-2 SingleQuots Supplements, Lonza). Cells were grown in T-75 flasks, coated with 2 μg/cm^2^ of poly-l-lysine for HBVP and HA. The growth medium was changed every two days. For experimental use, cells were grown until 90% confluence before subculturing; HA and HBVP were maintained between passages p1 and 4 and hCMEC/D3 cells were used for 10 passages. For the generation of BBB organoids, EGM-2 medium without VEGF was used, and named hereinafter Organoid Medium (OM).

### BBB organoid generation

HA, HBVP and hCMEC/D3 were detached by 0.05% trypsin/EDTA (ThermoFisher Scientific) and resuspended in warm OM. The concentration of each cell type was determined using the Countess™ automated cell counter (Invitrogen). The cells were resuspended at the appropriate concentration to target 1000 cells per microwell in a 1:1:1 ratio (i.e. a total of 3000 cells per microwell) in a seeding volume of 60 μL per well. 1 mL of media was added after 20 min. The cells were grown in a humidified incubator at 37 °C with 5% CO_2_ for 48 h to allow self-assembly of the multicellular organoids.

### Imaging and size analysis of BBB organoids

The assembled multicellular BBB organoids were imaged for size analysis after 48 h on a Nikon Eclipse Ti-E fully motorized microscope (Nikon) by automatically stitching 4 × 4 field of view images using a CFI Plan Fluor DL 4 ×  N.A. 0.13, W.D. 16.4 mm, PH-L air objective. All bright field images were analyzed and quantified using a custom-made Python script to estimate the diameter and size homogeneity of individual organoids.

### Immunofluorescence staining of BBB organoids

To specifically detect the localization of astrocytes within BBB organoids, HA were pre-labelled with CellTracker Red CMTPX at 10 µM in AGM for 15 min before organoid generation (ThermoFisher Scientific). Multicellular BBB organoid arrays were fixed in 4% paraformaldehyde (PFA) for 45 min at room temperature. Samples were washed thoroughly with PBS and were permeabilized and blocked with 0.6% Triton-X + 10% donkey serum in PBS for 1 h at room temperature. Organoids were then transferred to Eppendorf protein LoBind tubes for staining. All antibodies used in this study are reported in Table 1. Primary antibodies were then added in dilution buffer (0.1% Triton-X + 10% Donkey serum in PBS) and incubated 1 h at room temperature or overnight at 4 °C. Organoids were washed thoroughly in washing buffer (0.1% Triton-X in PBS) and incubated 1 h at room temperature or overnight at 4 °C with the respective species-specific fluorescently-labelled antibodies and DAPI (1 μg/mL, Sigma-Aldrich). Finally, the samples were washed again in the washing buffer, transferred to cover glasses, and mounted with Fluoromount (Electron Microscopy Science). Samples for qualitative evaluation of marker localization were imaged using a Leica SP5 confocal microscope using an HCX PL APO CS 40×/1.3 oil objective (Leica).

**Table 1:** List of antibodies

Antigen	Antibody type	Application	Manufacturer	Catalog number
Beta Actin	Monoclonal mouse IgG1 clone # 937215	WB	R&D Systems	MAB8929
Cas9	Monoclonal mouse IgG1	IF	ThermoFisher Scientific	MA1-202
Caveolin 1	Polyclonal rabbit IgG	IF	ThermoFisher Scientific	PA5-17447
Clathrin Heavy Chain	Polyclonal rabbit IgG	WB	ThermoFisher Scientific	PA5-84404
Human IgG (H + L)	Alexa Fluor^®^ 488 coupled goat AffiniPure Fab Fragment	IF	Jackson Immunoresearch	109-547-003
NG2	Polyclonal rabbit IgG	IF	Sigma-Aldrich	AB5320
Mouse IgG (H + L)	Alexa Fluor^®^ 488 coupled goat AffiniPure Fab Fragment	IF	Jackson Immunoresearch	115-547-003
Mouse IgG (H + L)	Alexa Fluor^®^ 594 coupled goat AffiniPure Fab Fragment	IF	Jackson Immunoresearch	115-587-003
P-gp	Monoclonal mouse IgG2a	IF	Biolegend	919403
Rabbit IgG (H + L)	Alexa Fluor^®^ 488 coupled donkey AffiniPure Fab Fragment	IF	ThermoFisher Scientific	711-547-003
TfR	Monoclonal rabbit IgG	WB	Cell Signaling Technology	13113S
ZO-1	Monoclonal mouse IgG1 clone 1A12	IF	ThermoFisher Scientific	33-9100

### Live/dead assay

After 48 h of assembly, BBB organoids were labelled with calcein-AM and ethidium homodimer-1 according to the manufacturer’s protocol (Live/DEAD Viability/Cytotoxicity Kit for mammalian cells, ThermoFisher Scientific) and were imaged using a Nikon Eclipse Ti-E fully motorized microscope (Nikon).

### Dextran permeability assay

Multicellular BBB organoid arrays were formed for 48 h and were incubated with 100 μg/mL FITC or 100 μg/mL FITC-Dextran of different molecular weight (3, 4, 40 and 70 kDa, Sigma-Aldrich and ThermoFisher Scientific) in Organoid Medium for 4 h at 37 °C with 5% CO_2_. Organoids were washed 3 times with PBS, and fixed in 4% PFA for 30 min at room temperature. The organoids were transferred to Eppendorf protein LoBind tubes and stained with DAPI (1 μg/mL, Sigma-Aldrich). Finally, organoids were washed with washing buffer (0.1% Triton-X in PBS), transferred to cover glasses, and mounted with Fluoromount (Electron Microscopy Science). Organoids were imaged using a Leica SP5 confocal microscope using an HCX PL APO CS 40×/1.3 oil objective (Leica). The organoid core was defined as the volume starting at 25 μm depth from the bottom of the organoid as visualized in an orthogonal cross-section. Z-stacks covering a total depth of 3.5 μm were acquired for quantification. Quantification of organoid permeability to FITC-dextran was performed using a custom-made automated Fiji [32] script that segments individual organoids and measures the mean fluorescence intensity of the maximum intensity projection within 75% of the cross-section area at the spheroid core. The code for the script is available at https://github.com/phagozyt/Fiji/blob/fb365d7c1275a6b013dc32b982504dfef9653d42/MIP75ROI.

### Transcytosis assay with BBB organoid arrays

After 48 h of assembly, BBB organoid arrays were incubated with custom-made humanized monovalent antibodies targeting either the human or the mouse transferrin receptor [2] and a non-targeting human IgG as a control in OM for 4 h at 37 °C with 5% CO_2_. To estimate the kinetics of transcytosis, BBB organoid arrays in separate wells were incubated with 100 nM of humanized monovalent antibodies against the human transferrin receptor for 15, 30, 60, 120, 240 or 360 min. After incubation, BBB organoids were washed thoroughly 6 times for 5 min with pre-warmed OM in the incubator, fixed with 4% PFA, processed for immunostaining and imaged for quantitative analysis as described above. For high-resolution analysis of localization of molecules within organoids, samples were imaged with a HCX PL APO lambda blue 63X/1.40 NA objective.

### Gene editing in BBB organoids

Ready-to-use lentiviral particles were purchased from Sigma-Aldrich. hCMEC/D3 cells were transduced first by lentiviral particles expressing Cas9 and blue fluorescent protein (BFP) as a transduction marker (LVCAS9BST, Sigma-Aldrich). Cas9-expressing cells were then selected with blasticidin antibiotic at 50 μg/mL for 6 days (Life technologies). Cas9-expressing hCMEC/D3 cells were each transduced separately by lentiviral particles expressing sgRNAs and selected by puromycin resistance at 10 μg/mL for 6 days (Life technologies). sgRNA sequences are listed in Table 2.

**Table 2:** List of sgRNAs

sgRNA name	Gene ID	Sanger Clone ID	PAM sequence	DNA target sequence
TFRC sgRNA	TFRC	HS5000003796	CGG	TGATCATAGTTGATAAGAACGG
CAV1 sgRNA	CAV1	HS0000173752	CGG	GACGTAGATGGAATAGACA
CLTC sgRNA	CLTC	HS5000032727	TGG	TAACTACAGGAAGTCGACTTGG

To generate monoclonal KO cell populations, Cas9-gRNA-expressing hCMEC/D3 were sorted (FACSAria II, BD Biosciences) using BFP into single clones and subsequently expanded.

### Western blot analysis

Whole cell lysates from hCMEC/D3 were prepared in RIPA lysis buffer (ThermoFisher Scientific) supplemented with cOmplet Protease Inhibitor Cocktail (Roche). Lysates were clarified by centrifugation (20,000*g*, 10 min, 4 °C) and protein concentrations were determined using the BCA protein assay kit (ThermoFisher Scientific). The same amount of protein (1.2 μg) was separated and analyzed for protein level using the 12–230 kDa Wes Separation Module with the Wes system (Protein Simple). All primary antibodies used are reported in Table 1. Secondary antibodies were used following the manufacturer recommendations (Protein Simple).

### Transferrin uptake

hCMEC/D3 cells were grown for 48 h on 24-well glass bottom plates (Mattek) pre-coated with collagen I (250 μg/mL, Sigma-Aldrich). To examine transferrin uptake, cells were incubated at 37 °C with 5% CO_2_ with Alexa Fluor 488–conjugated transferrin (25 μg/mL, ThermoFisher Scientific) for 30 min. Cells were quickly washed with PBS and fixed in 4% PFA for 20 min at room temperature. Simultaneous sample blocking and permeabilization were performed using 4% gelatin from cold water fish skin (Sigma-Aldrich) and 0.1% saponin (Sigma-Aldrich)  in PBS for 10 min at room temperature. Cells were then incubated with phalloidin atto-565 (200 pM, Sigma-Aldrich) and DAPI (1 μg/mL, Sigma-Aldrich) for 30 min at room temperature and washed 3 times with PBS. Cells were imaged with a DMi8S Leica widefield microscope using an HC PL APO 100×/1.47 oil objective. Quantification of total vesicle fluorescent intensity was made using MotionTracking as previously described [33, 34].

### Statistics

All values from quantifications are shown as box plots with median and interquartile ranges with lines representing the 5th and 95th percentiles. Statistical comparisons between multiple groups were performed by one-way analysis of variance (ANOVA), followed by Dunnett’s post hoc test. Values were considered to be significantly different when p < 0.05. Statistical analysis and plotting of data were performed with GraphPad Prism Software version 8.

## Results

### Assembly of BBB organoids in hydrogel microwell arrays

**Fig. 1:**
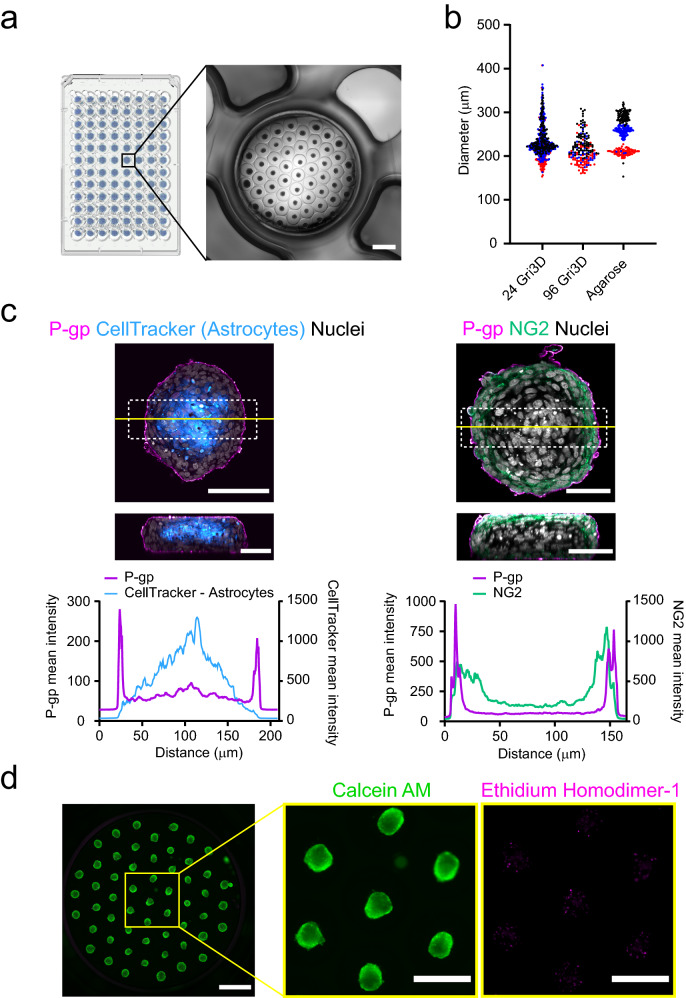
High-throughput blood–brain barrier organoid formation with microwell hydrogel arrays.** a** Schematic of microwell plates in 96-well plate format and representative phase contrast image of a GRi3D organoid array within a single well. Scale bar, 1 mm. **b** Quantification of blood–brain barrier organoid diameter in GRi3D microwell arrays within 24- or 96-well plate compared to organoids grown on agarose substrate. Each dot represents a single organoid, while different colors represent independent experiments. **c** Representative images of glass-mounted blood–brain barrier organoids labelled with cell type-specific markers showing the spatial distribution of brain endothelial cells (P-gp, magenta), pericytes (NG2, green) and astrocytes (CellTracker Red, cyan) within blood–brain barrier organoids grown in Gri3D arrays. Nuclei are labelled with DAPI (grey). Upper panels show single confocal sections below the spheroid surface. Scale bar, 100 μm. The yellow line shows the position of the orthogonal cross-section shown in the lower panel. Scale bar, 50 μm. Graphs show the mean line profile intensity fluorescence of each cell-type marker within organoids. Distance in μm is measured from left to right across the ROI shown within the dotted box. **d** Representative fluorescent image of a live/dead staining showing the viability of blood–brain barrier organoids grown on GRi3D arrays. Calcein AM in green labels live cells whereas ethidium homodimer-1 in magenta labels dead cells. Scale bar, 1 mm. Images on the right show a higher magnification of the boxed area. Scale bar 500 μm

To scale up the generation of BBB organoids, we used micropatterned hydrogel Gri3D plates, which allow the generation of thousands of organoids per experiment (Fig. 1a) [31]. Specifically, we co-cultured primary human astrocytes (HA) and human brain vascular pericytes (HBVP) with immortalized human brain cerebral microvascular endothelial cells (hCMEC/D3) in U-bottom microwell hydrogel-based inserts placed on custom-made 96-well plates. All three cell types were mixed in a 1:1:1 ratio and seeded on the hydrogel microcavity arrays to get a total average of 3000 cells per microcavity. After 48 h, cells formed compact homogeneous organoids throughout the plate at predefined locations and on the same focal plane (Fig. 1a). To assess the reproducibility of organoid arrays between experiments, we analyzed the diameter of BBB organoids generated in GRi3D microwell arrays and compared it to the previously reported method of organoid formation on agarose substrate [30]. In both cases the average diameter was comparable (220–250 µm) and in agreement with previously reported data [24, 26] (Fig. 1b). The diameter of BBB organoids in arrays showed higher intra-experiment variability (SD between 23 and 27) compared to agarose (SD between 6 and 22). However, organoid diameter in Gri3D plates was highly reproducible across 3 independent experiments, whereas in agarose the diameter varied up to 40% (Fig. 1b). Importantly, the average BBB organoid size was not affected by the specific Gri3D plate format, as we observed the same results using hydrogels in 24-well plates with the same microwell diameter (Fig. 1b). This demonstrates that BBB organoid assembly can be upscaled using larger arrays without affecting its reproducibility.

Next, we confirmed self-assembly and patterning of individual cell types into well-organized neurovascular structures by performing immunofluorescence stainings on compact BBB organoids grown in microwell arrays. We used a combination of cell-labelling and cell-specific markers to identify each cell type in the model; endothelial cells were tested for P-gp expression and pericytes for neural/glial antigen 2 expression (NG2). Astrocytes were labelled with CellTracker Red before assembly into BBB organoids. After 48 h of culture, we found that astrocytes accumulated within the core of the spheroid whereas brain endothelial cells (BECs) formed a continuous cellular layer at the surface of the organoids (Fig. 1c). A layer of pericytes formed between BECs in the surface and astrocytes located in the core of the organoids (Fig. 1c). This specific multicellular self-organization resembles the native cell–cell interactions within the BBB in vivo and is consistent with previous BBB organoid protocols, which used high-resolution imaging to show expression of BBB markers [24–26, 30]. Finally, we examined whether the high number of BBB organoids within the limited media volume in a single well would affect cell viability after 48 h of assembly. We labelled the BBB organoids with calcein-AM and ethidium homodimer-1 to detect viable and dead cells. We found that, under these conditions, the vast majority of cells in BBB organoids were viable and only a few dead cells were scattered across the organoid (Fig. 1d). Therefore, micropatterned hydrogel arrays offer a suitable alternative for reproducible growth of self-assembled and viable BBB organoids at a large scale.

### Antibody receptor-mediated transcytosis in BBB organoid arrays

**Fig. 2:**
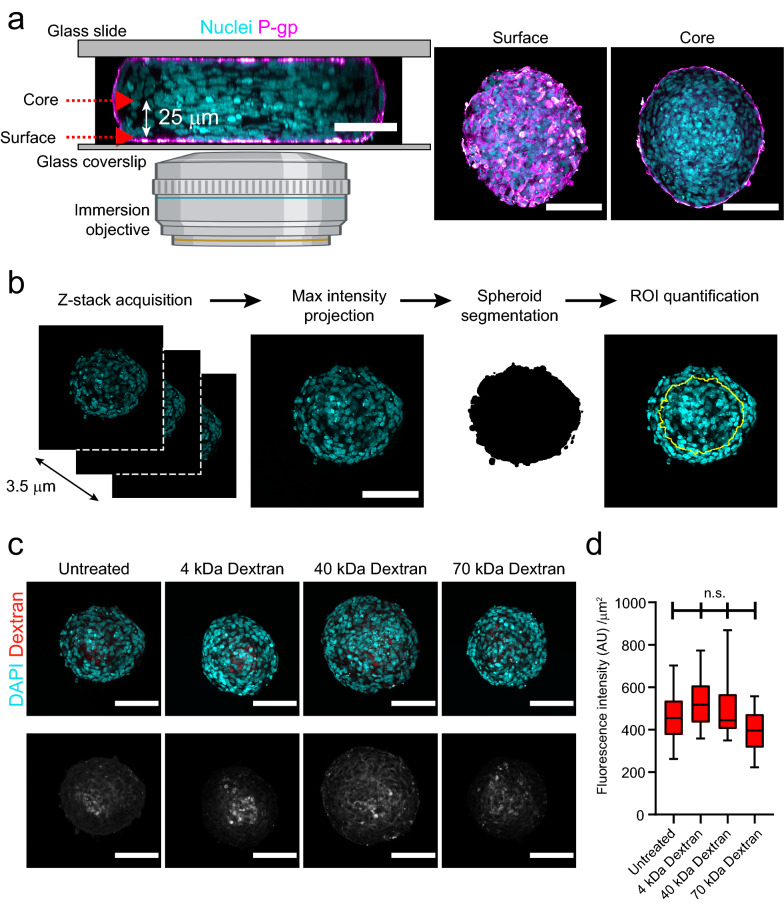
Standardized imaging workflow to assess transport into blood–brain barrier organoids.** a** Scheme and image showing the morphology of a blood-brain barrier organoid mounted on a glass slide and the relative position of imaging planes for experimental acquisition (left). Scale bar, 50 μm. Panels on the right show representative confocal images acquired at the organoid surface and core positions. Scale bar, 100 μm. In all images, brain endothelial cells are labelled with an anti-P-gp antibody (magenta) and are distributed at the organoid surface. Nuclei are labelled with DAPI (cyan). Schematic created with BioRender. **b** Scheme representing the Z-stack acquisition and analysis process (maximum intensity projection, segmentation and ROI definition) to quantify fluorescent molecules within the organoid core. In all images, nuclei are labelled with DAPI (cyan). Scale bar, 100 μm. **c** Representative confocal images of blood–brain barrier organoids incubated with different molecular weight Dextrans for 4 h. The upper images show an overlay of Dextran (red) and nuclei labelled with DAPI (cyan). The intensity of the lower images was scaled to visualize the background intensity in the Dextran channel (grey). Scale bars, 100 μm. **d** Quantification of Dextran fluorescence intensity within blood–brain barrier organoids. Graph shows boxplots with interquartile ranges and median. Lines show the 5th and 95th percentiles. Differences between treatments were not statistically significant (p = 0.51) as evaluated by the non-parametric Friedman test of 50 organoids per condition in n = 4 independent experiments

Next, we designed a standardized imaging workflow to estimate large molecule transport across organoids. We imaged BBB organoids mounted on glass slides to increase image resolution by the use of immersion objectives with high numerical aperture. In this setup, organoids were flattened between the glass slide and coverslip, which allowed imaging across the full 3D volume (Fig. 2a). Mounting occurred after chemical fixation and all functional assays (i.e. incubation with compounds) were performed using intact BBB organoids directly in hydrogel arrays. Importantly, mounting did not substantially damage organoids as cell layering in mounted BBB organoids (Fig. 1c) was comparable to layering observed in free-floating organoids [24, 26]. We used P-gp expression to identify and distinguish the organoid surface from the organoid core (Fig. 2a). We acquired all subsequent images at a penetration depth of at least 25 μm from the organoid surface. We reduced acquisition time by capturing a limited confocal volume (3.5 μm in z) within the core of each individual organoid. We analyzed these imaging data sets with a Fiji script that automatically segments BBB organoids and quantifies fluorescence intensity at the center of the organoid cross section (Fig. 2b). To test this accelerated imaging workflow and automated analysis, we first assessed the barrier function of BBB organoid arrays by incubating organoids with fluorescently-labelled dextran of different molecular weights (4, 40 and 70 kDa) or non-conjugated FITC (389 Da). In agreement with previous findings, we could not detect FITC-dextran within the organoid core, and the residual fluorescent signal was comparable with autofluorescence observed in non-treated BBB organoids (Fig. 2c). In contrast, non-conjugated FITC molecules accumulated within the core of BBB organoids (Additional file 1: Figure S1a), likely reflecting paracellular permeability across BECs. Importantly, FITC-dextran accumulated in organoids lacking BECs  (Additional file 1: Figure S1a), showing that BECs are sufficient to restrict the permeability of Dextran in BBB organoids. These qualitative observations were confirmed by the automatic quantification of a larger population of BBB organoids across independent experiments with the image analysis script (Fig. 2d; Additional file 1: Figure S1b). The barrier function of BBB organoids is further supported by the expression and localization of tight junction protein ZO-1 at the spheroid surface (Additional file 1: Figure S2). These results show that BBB organoid arrays form tight junctions and maintain the low paracellular permeability to macromolecules reported in other in vitro models [24–26, 30, 35].

**Fig. 3:**
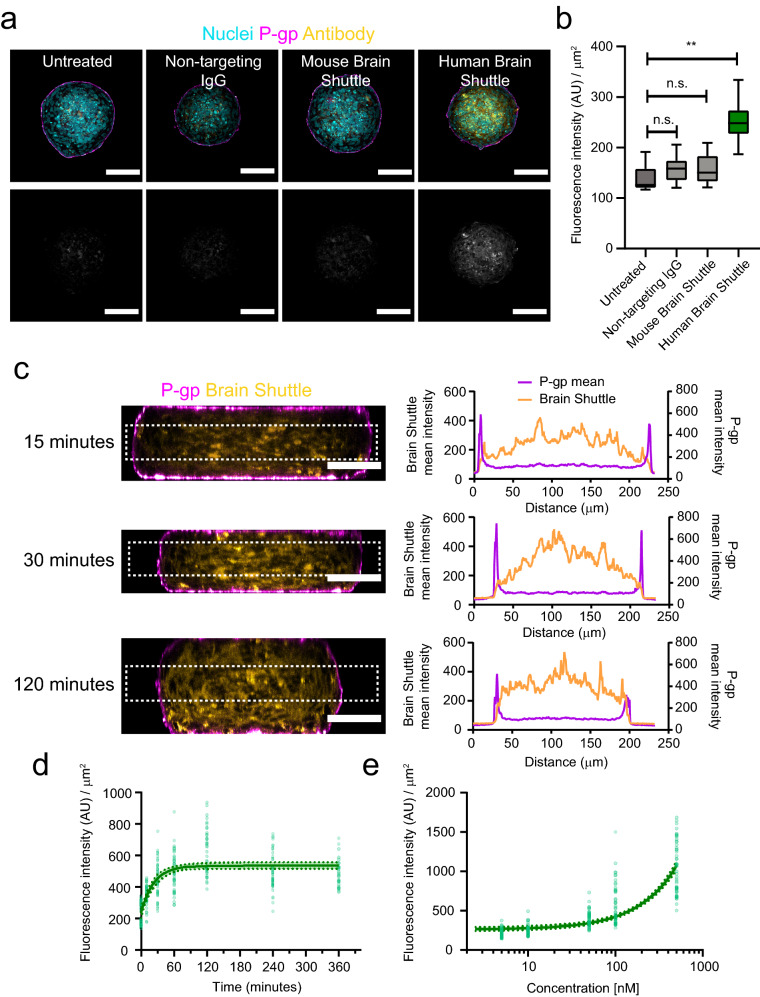
Receptor-mediated transcytosis of a human Brain Shuttle antibody in blood–brain barrier organoids. **a** Representative confocal images of blood–brain barrier organoids after incubation with different antibody constructs for 4 h. The upper images show an overlay of brain endothelial cells labelled with an anti-P-gp antibody (magenta), antibody constructs labelled with anti-human IgG Fab fragments (yellow) and nuclei labelled with DAPI (cyan). The intensity of the lower images was scaled to visualize the background intensity in the antibody channel (grey). Scale bars, 100 μm. **b** Quantification of antibody construct transcytosis into blood–brain barrier organoids. Graph shows boxplots with interquartile ranges and median. Lines show the 5th and 95th percentiles. Differences between non-treated organoids and non-targeting IgG or mouse brain shuttle were not statistically significant (p > 0.6) whereas differences between human Brain Shuttle and non-treated control were statistically significant (**p = 0.002) as evaluated by one-way ANOVA followed by Dunnett’s test for multiple comparisons of 60 organoids per condition in n = 3 independent experiments. **c** Representative orthogonal cross-sections assembled from confocal planes spanning the whole volume of organoids incubated with human Brain Shuttle for 15, 30 or 120 min. The images on the left show the maximum projection of 10 orthogonal planes located at least 25 μm below the organoid surface. Brain endothelial cells are labelled with an anti-P-gp antibody (magenta), antibody constructs are labelled with anti-human IgG Fab fragments (yellow). Scale bars, 50 μm. The graphs on the right show the mean line profile intensity of human Brain Shuttle (yellow) or P-gp (magenta) fluorescence within organoids. Distance in μm is measured from left to right across the ROI shown within the dotted box. **d** Time course of human Brain Shuttle transcytosis into the core of blood–brain barrier organoids reconstructed from organoid populations fixed with PFA at different time points. **e** Dose–response of human Brain Shuttle transcytosis into the core of blood–brain barrier organoids. In **d** and **e** points represent individual organoids. The solid lines show the best nonlinear (**d**) or linear (**e**) fit of the experimental data. The dotted lines and colored area show the 95% confidence interval of the curve estimation

Next, we evaluated the BBB organoid arrays as a screening platform for receptor-mediated transcytosis with this imaging workflow. We used a monovalent antibody against the extracellular domain of the human transferrin receptor (TfR). We refer to this antibody as human Brain Shuttle [2]. We incubated BBB organoid arrays with 100 nM of non-labelled antibody and detected it after 4 h with fluorescently-labelled anti human IgG-specific Fab fragments. As negative controls, we used a non-targeting human IgG and a monovalent antibody against the mouse TfR that does not cross-react with the human receptor. We refer to this mouse-specific TfR antibody as mouse Brain Shuttle. Under these conditions, we did not detect either the non-targeting IgG or the mouse Brain Shuttle within BBB organoids. In contrast, we detected substantial accumulation of human Brain Shuttle within the organoid core (Fig. 3a, b). To confirm that the human Brain Shuttle crossed the endothelial cell layer at the surface of organoids, we visualized the antibody distribution after incubation for different time points in orthogonal cross-sections of PFA-fixed organoids. At 15 min, we observed human Brain Shuttle signal at the organoid core (Fig. 3c). At 30 min, the overall intensity increased throughout the organoid core and did not increase further at 120 min (Fig. 3c). Together, these observations show that the Brain Shuttle signal does not originate solely from binding and/or uptake at the organoid surface but reflects transport across the endothelial cell layer into the organoid core. We confirmed time- and dose-dependent transcytosis of human Brain Shuttle by quantifying more than 50 individual BBB organoids per condition (Fig. 3d, e). Overall, these data show that BBB organoid arrays combined with this optimized imaging and analysis workflow are a suitable platform for identifying and characterizing antibodies that undergo receptor-mediated transcytosis across the human BBB.

### Dissecting the molecular regulation of transcytosis with BBB organoid arrays

**Fig. 4:**
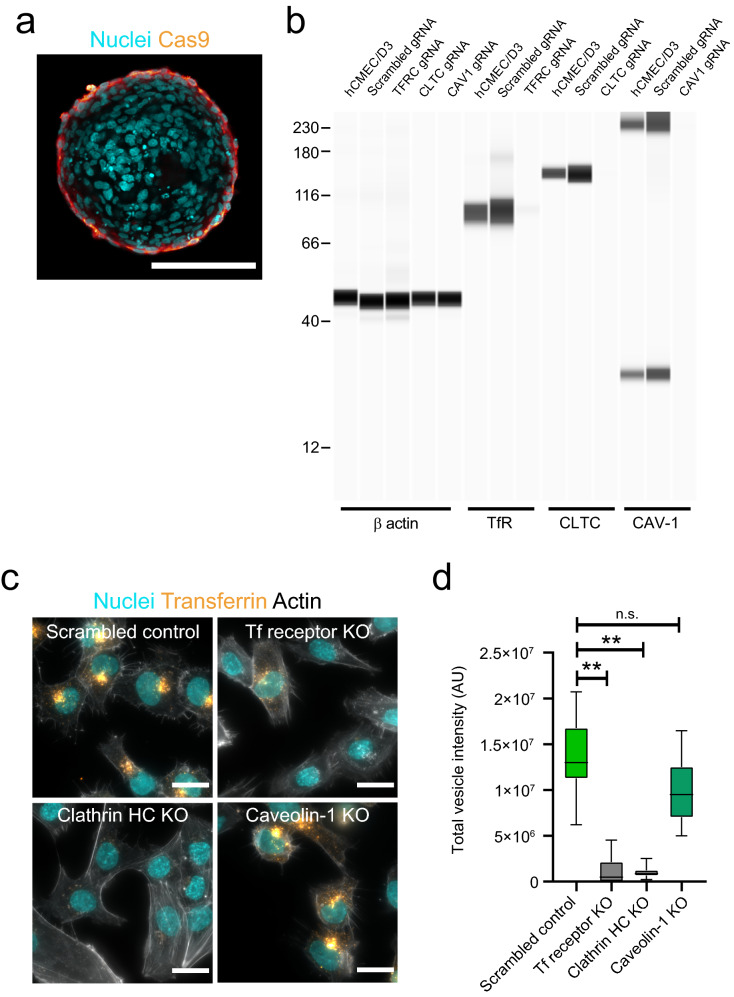
Establishment and characterization of Cas9 brain endothelial cell lines. **a** Representative confocal image acquired at the core of a blood-barrier organoid assembled with brain endothelial cells expressing Cas9 (orange). Cas9 brain endothelial cells localize only at the periphery of blood–brain barrier organoids. Nuclei labelled with DAPI (cyan). Scale bar, 100 μm. **b** Representative Western blot image showing the expression of Transferrin receptor, clathrin heavy chain or caveolin-1 in hCMEC/D3 brain endothelial cells expressing Cas9 and transduced with either scrambled gRNA or gRNA against the target gene. β actin expression is shown as a reference control gene. **c** Representative fluorescent images of hCMEC/D3 brain endothelial Cas9 or knockout cells after incubation with fluorescently labelled transferrin (yellow) for 30 min. Actin is labelled with phalloidin (grey) to visualize cell contours and nuclei are labelled with DAPI (cyan). Scale bars, 20 μm. **d** Quantification of transferrin internalization in hCMEC/D3 Cas9 or knockout cells. Graph shows boxplots with interquartile ranges and median. Lines show the 5th and 95th percentiles. Differences between the scrambled control and transferrin receptor or clathrin heavy-chain knockout cells were statistically significant (**p = 0.018) whereas the difference between the scrambled control and caveolin-1 knockout cells was not statistically significant (p = 0.418). Comparisons were evaluated by one-way ANOVA followed by Dunnett’s test for multiple comparisons of ~ 400 single cells per condition in n = 2 independent experiments

As BBB organoid arrays enable robust and sensitive transport assays, we further investigated the mechanisms of receptor-mediated transcytosis with CRISPR-based gene editing. In order to interrogate the role of specific genes on transcytosis across the BBB, we first generated a human brain endothelial cell line (hCMEC/D3) stably expressing Cas9. Importantly, this Cas9-expressing cell line maintained its capacity to self-organize at the surface of BBB organoids (Fig. 4a). To validate the use of CRISPR-based gene editing on BBB organoid arrays and as a proof-of-concept, we selected genes with well-characterized functions in transferrin transport and/or endocytosis: TfR, clathrin heavy chain and caveolin-1 [36–38]. We used a non-targeting scrambled sgRNA as a negative control. To functionally characterize these cell lines we analyzed protein expression and quantified transferrin uptake, a well-established assay to evaluate perturbations to endocytosis [33]. Western blot analysis showed that target protein expression was substantially reduced in all three cell lines compared to the parental and control cells (Fig. 4b). In the internalization assay using hCMEC/D3 cells grown on glass coverslips, we observed accumulation of transferrin at the perinuclear region in control cells. On the other hand, transferrin internalization was abrogated in both TfR and clathrin heavy chain knockout cells (Fig. 4c, d), in agreement with the essential role of both genes for transferrin uptake [38, 39]. It should be noted that we observed across multiple experiments a fraction of TfR knockout cells that still internalized transferrin, likely reflecting either a small heterozygous population or aggregate, non-functional transferrin taken up by non-specific endocytosis. In contrast, the extent of transferrin internalization and its intracellular localization was not changed in caveolin-1 knockout cells compared to the control (Fig. 4c, d). This result confirms that the primary pathway for transferrin internalization in brain endothelial cells is via clathrin-mediated endocytosis [16].

**Fig. 5:**
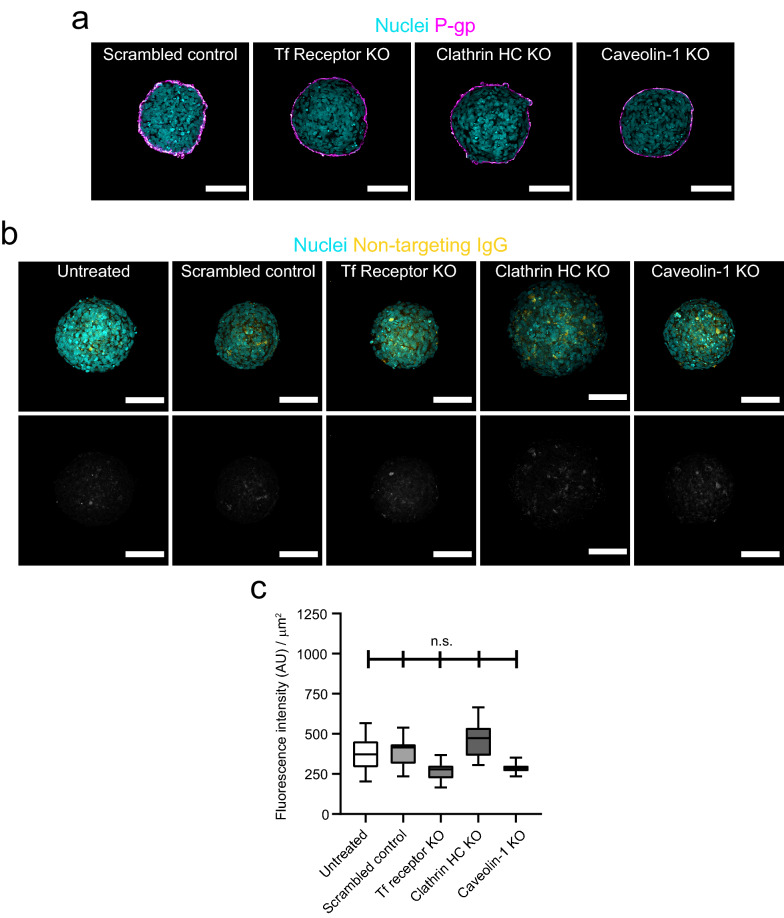
CRISPR/Cas9 gene editing in blood–brain barrier organoids does not disrupt barrier function.** a** Representative confocal images of blood–brain barrier organoids assembled with hCMEC/D3 Cas9/Scrambled gRNA control or knockout cells. Brain endothelial cells are labelled with an anti-P-gp antibody (magenta) and are homogeneously distributed at the organoid surface. Nuclei are labelled with DAPI (cyan). Scale bar, 100 μm. **b** Representative confocal images of blood–brain barrier organoids incubated with a non-targeting human IgG for 4 h. The upper images show an overlay of human IgG signal (yellow) and nuclei labelled with DAPI (cyan). The intensity of the lower images was scaled to visualize the background intensity in the antibody channel (grey). Scale bar, 100 μm. **c** Quantification of IgG intensity within blood–brain barrier organoids. Graph shows boxplots with interquartile ranges and median. Lines show the 5th and 95th percentiles. Differences between treatments were not statistically significant (p = 0.344) as evaluated by one-way ANOVA of 30 organoids per condition in n = 2 independent experiments

**Fig. 6:**
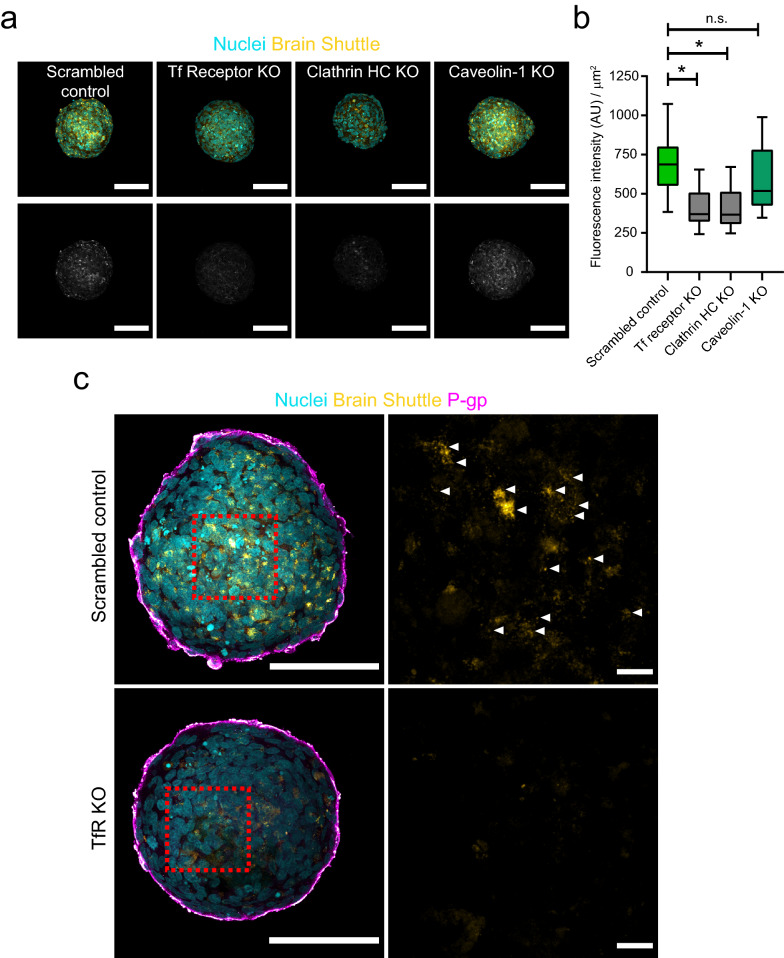
CRISPR/Cas9 gene editing in blood–brain barrier organoids to investigate the mechanisms of transcytosis.** a** Representative confocal images of blood–brain barrier organoids assembled with hCMEC/D3 Cas9 knockout cells incubated with a human Brain Shuttle antibody for 4 h. The upper images show an overlay of human IgG signal (yellow) and nuclei labelled with DAPI (cyan). The intensity of the lower images was scaled to visualize the background intensity in the antibody channel (grey). Scale bar, 100 μm. **b** Quantification of IgG intensity within blood–brain barrier organoids. Graph shows boxplots with interquartile ranges and median. Lines show the 5th and 95th percentiles. Differences between the scrambled control and transferrin receptor or clathrin heavy-chain knockout organoids were statistically significant (*p < 0.05) whereas the difference between the scrambled control and caveolin-1 knockout organoids was not statistically significant (p = 0.609). Comparisons were evaluated by one-way ANOVA followed by Dunnett’s test for multiple comparisons of ~ 50 organoids per condition in n = 3 independent experiments. **c** Representative confocal images of human Brain shuttle antibody distribution in control (upper panels) or transferrin receptor knock-out (lower panels) blood–brain barrier organoids treated as in **a**. Panels on the left show a low magnification image of an organoid. Scale bar, 100 μm. Panels on the right show a high magnification image of the boxed area. Arrowheads point to accumulation of Brain Shuttle signal in puncta and/or tubules. Scale bar, 10 μm

We next used each of these knockout cell lines to form BBB organoid arrays and assessed their impact on antibody receptor-mediated transcytosis. Similar to parental hCMEC/D3 or Cas9 cells, all knockout cell lines maintained their capacity to assemble at the surface of BBB organoids (Fig. 5a). Furthermore, we could not detect non-targeting IgG within the core of BBB organoids assembled with TfR, clathrin heavy chain or caveolin-1 knockout cells (Fig. 5b, c), strongly suggesting that these genes do not affect BBB paracellular permeability. Finally, we incubated knockout organoids with human Brain Shuttle to evaluate receptor-mediated transcytosis. In control organoids, human Brain Shuttle accumulated within the organoid core, whereas in TfR and clathrin heavy-chain knockout organoids the fluorescent signal was substantially reduced and comparable to background levels (Fig. 6a, b). Interestingly, we found that the human Brain Shuttle showed a clear punctate distribution in control organoids, likely reflecting endocytosis by astrocytes within the organoid core (Fig. 6c). Caveolin-1 knockout had no major impact on the transport of human Brain Shuttle into the organoid core (Fig. 6a, b). These data show that transcytosis of the human Brain Shuttle across the BBB is TfR-specific and requires clathrin but occurs independently of caveolin-1. Overall, our results demonstrate that BBB organoid arrays can be combined with CRISPR gene editing to interrogate the molecular mechanisms of transcytosis across the human BBB.

## Discussion

A major obstacle for accelerating the development of new brain targeting biologics is the lack of high-throughput in vitro BBB models that are translatable to humans. Here, we bridged this gap by using a novel bioengineering approach relying on hydrogel-based patterned microcavity arrays to grow homogeneous organoids [31, 40, 41]. Previous work established that BBB organoids recapitulate key properties of the BBB observed in vivo [25, 26, 30] and constituted a major advance in the field. Specifically, organoid assembly increased expression of receptors involved in transcytosis, transporters, and tight junction proteins compared to a monoculture of endothelial cells [24, 26, 27]. Furthermore, it was shown that BBB organoids assembled with hCMEC/D3 cells reproduced the expected permeability of angiopep-2 whereas the same cells grown in a transwell model failed to do so [26]. Altogether, these data strongly suggested that BBB organoids are a superior alternative to BECs grown in a transwell configuration. Nevertheless, we need to stress that the fact that non-conjugated FITC accumulated within BBB organoids (Figure S1) suggests that further optimization is needed to fully recapitulate the low permeability of the BBB observed in vivo for small molecules [42]. The model and workflow we developed confirms the key properties and functionality of BBB organoids and builds upon previous work by making substantial improvements to facilitate its widespread adoption for drug discovery. First, microwell arrays increased the BBB organoid yield more than 30 fold per experiment compared to previous protocols using agarose [26, 30] or hanging-drop culture [24, 25]. This yield increase is accompanied by a substantial reduction in experimental handling time, as hydrogel production can be automated [31] and replaces manual preparation of individual plates [30]. Second, BBB organoid formation in arrays was highly reproducible between independent experiments, whereas assembly in agarose resulted in inter-experimental variation of up to 40% in organoid size. Third, we used an automated image analysis script on confocal microscopy images to measure the accumulation of antibodies within organoids. This streamlined workflow enabled the measurement of nearly 10 times more organoids compared to previous studies [25, 30]. This larger data set allowed us to robustly estimate the kinetics of human Brain Shuttle transcytosis. Interestingly, accumulation of human Brain Shuttle in BBB organoids reached a steady-state around 60 min (Fig. 3d), which could be the result of receptor saturation or the balance between transcytosis and efflux/recycling [43, 44]. Via this workflow, various scenarios can be evaluated in more detailed mechanistic studies with the same in vitro platform. Fourth, the transport assay in BBB organoid arrays allows the characterization of native, non-labelled antibodies, whereas previous work measured BBB-transport of biologics using molecules that were covalently coupled with organic fluorophores [18, 26]. While detection of fluorescent conjugates with microscopy requires no additional sample preparation, it does require the prior chemical labelling of each one of the molecules in the test set, which may not be feasible during lead identification and optimization phases. On the other hand, a limitation of detecting non-labelled antibodies by immunofluorescent labeling as done in this study is that it prevents absolute quantification of the amount of transcytosed molecules. Therefore, quantifying permeability coefficients for direct comparison with in vivo observations as done in studies with microfluidic platforms [35, 45] is currently not possible with BBB organoids arrays. This could be overcome by using radiolabelled molecules or by performing antibody measurements from organoid lysates with analytical methods such as ELISA. However, unlike our approach of quantifying the 3D distribution of fluorescence intensity in organoids, these analytical methods cannot distinguish between antibodies bound at the surface and those that underwent transcytosis. Ultimately, this would overestimate the extent of transport across the BBB and yield false-positive hits during a screening campaign. Instead, we consider that combining single cell sorting with analytical methods as recently done in vivo [46] could be a more promising strategy for absolute quantification in organoid models in the future. Altogether, we consider that these improvements make BBB organoid arrays an optimal platform for drug discovery to identify biologics that can cross the BBB in a scalable manner. The use of high-throughput human in vitro models of the BBB will advance translatability of early discovery work into the clinic.

The reproducibility and high sensitivity of the transport assay in BBB organoid arrays make them a useful tool to dissect the molecular mechanisms of transcytosis. We showed that CRISPR-based gene editing can be combined with BBB organoid arrays to evaluate the role of individual genes in receptor-mediated transcytosis. Therefore, this platform will enable the execution of functional large scale screens to identify novel regulators of transcytosis. As a first proof-of-concept we focused on well-characterized genes and investigated caveolin-1 depletion as it has been proposed to be a key regulator of transcytosis across the BBB [20, 21, 47]. However, our data shows that clathrin, but not caveolin-1, is required for transferrin-receptor mediated transcytosis of a human Brain Shuttle. This finding supports the presence of multiple transcytosis pathways with different molecular machineries in brain endothelial cells [48]. Overall, we consider this approach will be useful to evaluate whether different formats (e.g. bispecific antibodies, single domain antibodies, multivalent coated particles) or receptors utilize the same transport pathways across the BBB.

## Conclusions

Human BBB organoid arrays are a high-throughput platform to generate homogeneous and reproducible BBB organoids that allow the identification and characterization of antibodies undergoing receptor-mediated transcytosis. We show here that combining high-throughput organoid analysis and gene editing technologies can lead to novel insights into the molecular regulation of transcytosis across the BBB. Implementation of transport assays with BBB organoid arrays will help accelerate the discovery and development of new brain targeting modalities.

## Supplementary Information

**Additional file 1:Figure S1**. a, Representative confocal images of blood-brain barrier organoids assembled with astrocytes, pericytes and brain endothelial cells (BBB) or with astrocytes and pericytes only (no BECs) incubated with different molecular weight Dextrans or free FITC for 4 hours. The upper images show an overlay of Dextran (red) and nuclei labelled with DAPI (cyan). The intensity of the lower images was scaled to visualize the background intensity in the Dextran channel (grey). Scale bars, 100 μm. b, Quantification of fluorescence intensity within blood-brain barrier organoids. Graph shows boxplots with interquartile ranges and median. Lines show the 5th and 95th percentiles. Differences between treatments were evaluated by Kruskal-Wallis test followed by Dunn’s test for multiple comparisons of at least 30 organoids per condition in n = 2 independent experiments. ***, p < 0.01. n.s., not significant. **Figure S2**. Representative confocal images of ZO-1 localization in a blood-brain barrier organoid. The images on the upper panel show an orthogonal cross-section assembled from confocal planes spanning the whole volume of a blood-brain barrier organoid. Scale bar, 50 μm. Panels on the bottom show representative confocal images acquired at the organoid surface and core positions. The panel on the bottom right shows a higher magnification image of the boxed yellow region. Scale bar, 50 μm. In all images, ZO-1 is shown in orange and DAPIlabelled nuclei are shown in cyan.

## Data Availability

The datasets generated and/or analyzed during the current study are available from the corresponding author upon reasonable request.

## Abbreviations

ALPL: Alkaline phosphatase
BBB: Blood–brain barrier
BEC: Brain endothelial cell
CRISPR: Clustered regularly interspaced short palindromic repeats
CNS: Central nervous system
EDTA: Ethylenediaminetetraacetic acid
ELISA: Enzyme-linked immunosorbent assay
FBS: Fetal bovine serum
FITC: Fluorescein isothiocyanate
GA-1000: Gentamicin sulfate-amphotericin
HA: Human astrocytes
HBVP: Human brain vascular pericytes
hCMEC/D3: Human cerebral microvascular endothelial D3 cells
hEGF: Human epidermal growth factor
hFGF-B: Human fibroblast growth factor B
IF: Immunofluorescence
NG2: Neural/glial antigen 2
Mfsd2a: Major facilitator superfamily domain-containing protein 2
PBS: Phosphate buffered saline
PEG: Polyethylene glycol
PGP: P-glycoprotein 1
R3-IGF-I: Recombinant insulin-like growth factor-I
TfR: Transferrin receptor
VEGF: Vascular endothelial growth factor
WB: Western blot
