# Supplementary material for: Investigating receptor-mediated antibody transcytosis using blood–brain barrier organoid arrays
Source: Fluids Barriers CNS. 2021 Sep 20;18:43. doi: 10.1186/s12987-021-00276-x (PMC8454074; doi:10.1186/s12987-021-00276-x)
Supplement: Supplementary file 1 — Additional file 1:Figure S1. a, Representative confocal images of blood-brain barrier organoids assembled with astrocytes, pericytes and brain endothelial cells (BBB) or with astrocytes and pericytes only (no BECs) incubated with different molecular weight Dextrans or free FITC for 4 hours. The upper images show an overlay of Dextran (red) and nuclei labelled with DAPI (cyan). The intensity of the lower images was scaled to visualize the background intensity in the Dextran channel (grey). Scale bars, 100 μm. b, Quantification of fluorescence intensity within blood-brain barrier organoids. Graph shows boxplots with interquartile ranges and median. Lines show the 5th and 95th percentiles. Differences between treatments were evaluated by Kruskal-Wallis test followed by Dunn’s test for multiple comparisons of at least 30 organoids per condition in n = 2 independent experiments. ***, p < 0.01. n.s., not significant. Figure S2. Representative confocal images of ZO-1 localization in a blood-brain barrier organoid. The images on the upper panel show an orthogonal cross-section assembled from confocal planes spanning the whole volume of a blood-brain barrier organoid. Scale bar, 50 μm. Panels on the bottom show representative confocal images acquired at the organoid surface and core positions. The panel on the bottom right shows a higher magnification image of the boxed yellow region. Scale bar, 50 μm. In all images, ZO-1 is shown in orange and DAPIlabelled nuclei are shown in cyan. [file 12987_2021_276_MOESM1_ESM.pdf]

Additional File 1

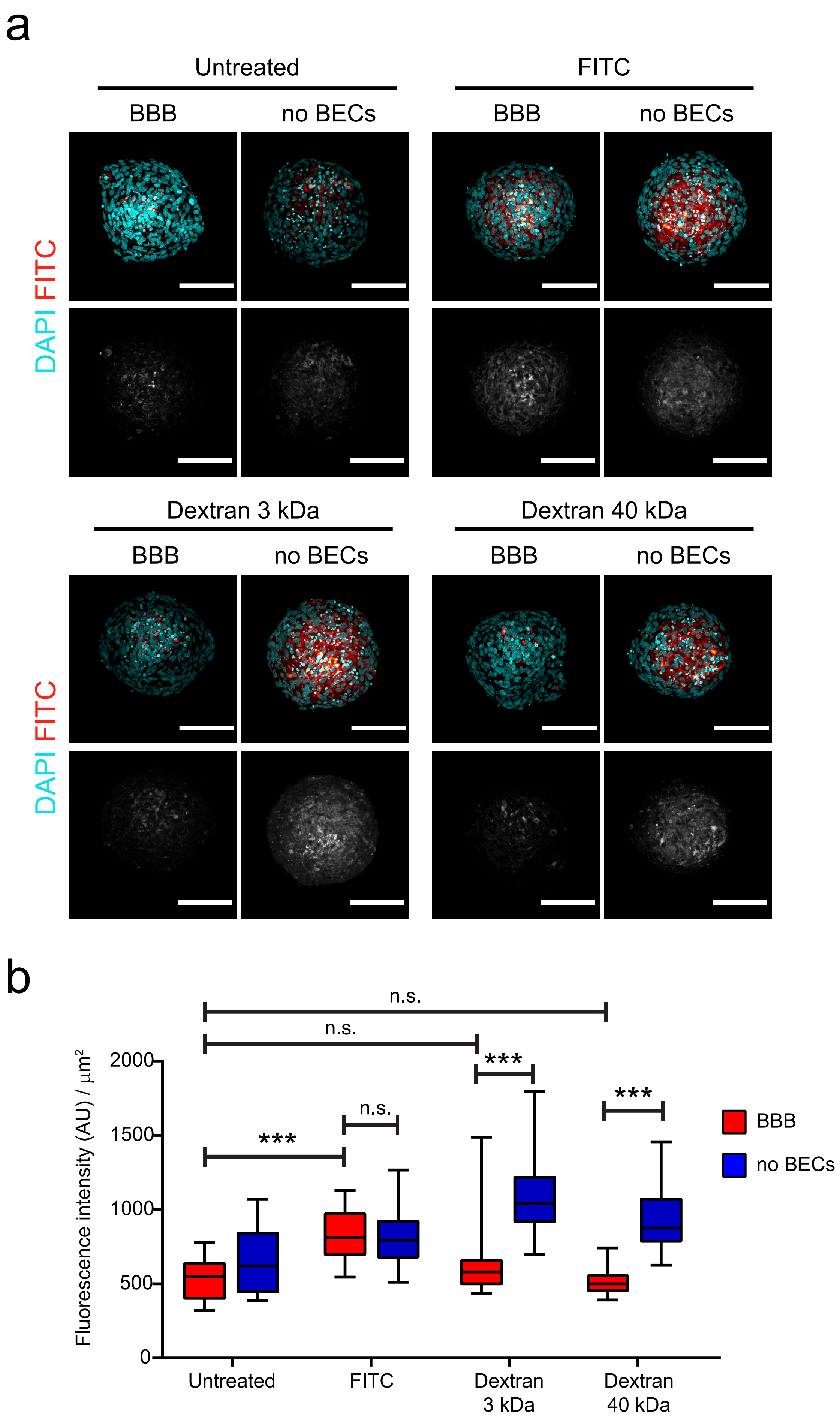

Figure S1. a, Representative confocal images of blood-brain barrier organoids assembled with astrocytes, pericytes and brain endothelial cells (BBB) or with astrocytes and pericytes only (no BECs) incubated with different molecular weight Dextrans or free FITC for 4 hours. The upper images show an overlay of Dextran (red) and nuclei labelled with DAPI (cyan). The intensity of the lower images was scaled to visualize the background intensity in the Dextran channel (grey). Scale bars, 100 μm. **b,** Quantification of fluorescence intensity within blood-brain barrier organoids. Graph shows boxplots with interquartile ranges and median. Lines show the 5th and 95th percentiles. Differences between treatments were evaluated by Kruskal-Wallis test followed by Dunn's test for multiple comparisons of at least 30 organoids per condition in n = 2 independent experiments. ***, $p < 0.01$. n.s., not significant.

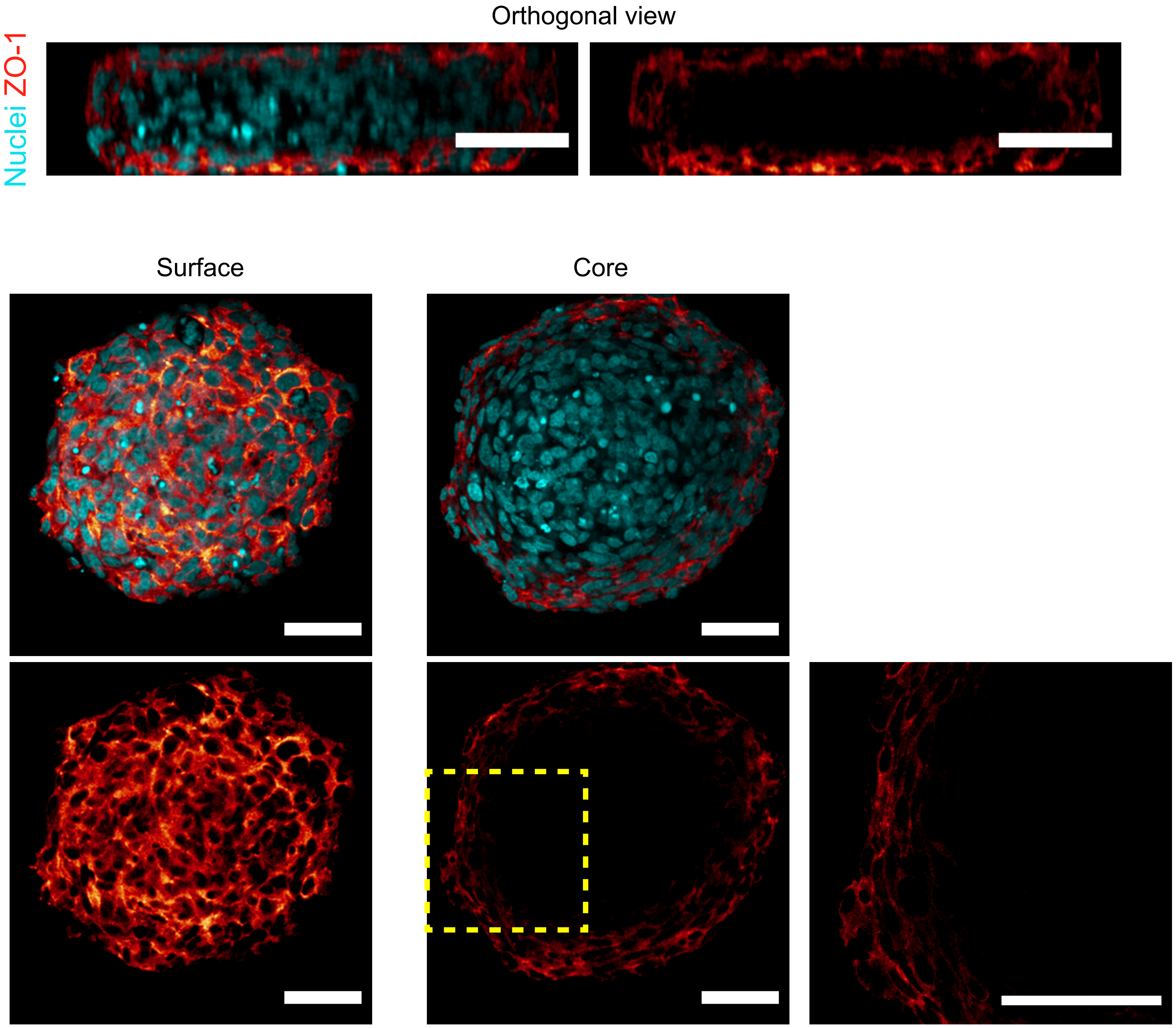

Figure S2. Representative confocal images of ZO-1 localization in a blood-brain barrier organoid. The images on the upper panel show an orthogonal cross-section assembled from confocal planes spanning the whole volume of a blood-brain barrier organoid. Scale bar, 50 µm. Panels on the bottom show representative confocal images acquired at the organoid surface and core positions. The panel on the bottom right shows a higher magnification image of the boxed yellow region. Scale bar, 50 µm. In all images, ZO-1 is shown in orange and DAPI-labelled nuclei are shown in cyan.
